# *KCNC2* variants of uncertain significance are also associated to various forms of epilepsy

**DOI:** 10.3389/fneur.2023.1212079

**Published:** 2023-06-09

**Authors:** Simone Seiffert, Manuela Pendziwiat, Ulrike B. S. Hedrich, Ingo Helbig, Yvonne Weber, Niklas Schwarz

**Affiliations:** ^1^Department of Human Genetics, University Hospital Ulm, Ulm, Germany; ^2^Department of Neurology and Epileptology, Hertie-Institute for Clinical Brain Research, University of Tübingen, Tübingen, Germany; ^3^Institute of Clinical Molecular Biology, Christian-Albrechts-University of Kiel, Kiel, Germany; ^4^Department of Neuropediatrics, University Medical Center Schleswig-Holstein, Christian-Albrechts-University, Kiel, Germany; ^5^Division of Neurology, Children’s Hospital of Philadelphia, Philadelphia, PA, United States; ^6^The Epilepsy NeuroGenetics Initiative (ENGIN), Children's Hospital of Philadelphia, Philadelphia, PA, United States; ^7^Department of Biomedical and Health Informatics (DBHi), Children’s Hospital of Philadelphia, Philadelphia, PA, United States; ^8^Department of Neurology, Perelman School of Medicine, University of Pennsylvania, Philadelphia, PA, United States; ^9^Department of Epileptology and Neurology, University of Aachen, Aachen, Germany

**Keywords:** potassium channel, developmental and epileptic encephalopathy, *KCNC2* electrophysiology, functional analysis, precision medicine

## Abstract

Recently, *de novo* variants in *KCNC2*, coding for the potassium channel subunit K_V_3.2, have been described as causative for various forms of epilepsy including genetic generalized epilepsy (GGE) and developmental and epileptic encephalopathy (DEE). Here, we report the functional characteristics of three additional *KCNC2* variants of uncertain significance and one variant classified as pathogenic. Electrophysiological studies were performed in *Xenopus laevis* oocytes. The data presented here support that *KCNC2* variants with uncertain significance may also be causative for various forms of epilepsy, as they show changes in the current amplitude and activation and deactivation kinetics of the channel, depending on the variant. In addition, we investigated the effect of valproic acid on K_V_3.2, as several patients carrying pathogenic variants in the *KCNC2* gene achieved significant seizure reduction or seizure freedom with this drug. However, in our electrophysiological investigations, no change on the behavior of K_V_3.2 channels could be observed, suggesting that the therapeutic effect of VPA may be explained by other mechanisms.

## Introduction

Epilepsy is one of the most common neurological disorders affecting approximately 1% of the world’s population (1). The implementation of next generation sequencing techniques has led to the identification of various ion channel genes as causes of neurological disorders, including epilepsy. Ion channels play a central role in neuronal excitability and neurotransmitter release, and their altered function appears to be a substantial factor in the etiology of genetic epilepsies. It is therefore not surprising that over time also more and more potassium channel genes have emerged as interesting candidates for epilepsies (2, 3). Variants in genes encoding potassium channels induce different forms of epilepsy, ranging from benign familial neonatal seizures (*KCNQ2/3*, encoding K_V_7.2/3) (4, 5) to severe developmental and epileptic encephalopathies (*KCNA2*, encoding K_V_1.2) (6, 7). In daily work, one major problem in the interpretation of exome sequencing is that many variants of unknown relevance for the phenotype are detected. Based on variant frequency, *in silico* prediction tools, the crystal structure of the protein and ACMG criteria (criteria for the interpretation of variants from the American college of medical genetics and genomics and the association for molecular pathology), variants can be defined as pathogenic, likely pathogenic or benign (8). Nevertheless, for many variants, the functional testing in respective *in vitro* or *in vivo* systems is not available.

Recently, we described variants in the *KCNC2* gene, encoding the potassium channel subunit K_V_3.2, as causative for several forms of epilepsy, including genetic generalized epilepsy (GGE) and developmental and epileptic encephalopathy (DEE) (9). K_V_3.2, a member of the Shaw-related (K_V_3) voltage-gated potassium channel subfamily, is expressed primarily in the cerebral cortex, hippocampus, and basal ganglia and plays an important role in modulating the action potential duration and neurotransmitter release (10, 11). In this work, we mainly wanted to test if variants in *KCNC2* that were defined as variants of uncertain significance (VUS) according to the ACMG criteria, also show a functional effect and can be associated to the epilepsy phenotype. We also tested one pathogenic variant that has not functionally characterized before. Therefore, we used *Xenopus laevis* oocytes as heterologous expression system. Additionally, we were interested in the effect of valproic acid, which was demonstrated to be particularly effective in some of the *KCNC2* cases described previously (9).

## Subjects and methods

### Patients

Previously, we described 18 patients with pathogenic or likely pathogenic *KCNC2* variants but functionally only characterized the clear pathogenic ones (9). Here, we focused on the functional consequences of variants that have been identified in four patients: p. (Ser333Thr) (S333T, VUS, phenotype: Dravet like syndrome), p. (Phe382Cys) (F382C, pathogenic, phenotype: MAE, myoclonic astatic epilepsy), p. (Ile465Val) (I465V, VUS, phenotype: focal epilepsy) and p. (Asn530His) (N530H, VUS, phenotype: GGE, genetic generalized epilepsy) in the *KCNC2* gene. Clinical data of these four patients have been previously described in more detail. Written informed consent was obtained from all participants in the study or by the legal representatives (9). Four variants were selected for functional analysis according to the associated phenotype, the location, and the pathogenic or uncertain significance character based on the ACMG criteria.

### Backbone and RNA preparation

Vector pcDNA3.1 (+) + insert *KCNC2* WT and the mutant clones (NM_139137: c.G998C/p.(Ser333Thr)/S333T; c.T1145G/p.(Phe382Cys)/F382C; c.A1393G/p.(Ile465Val)/I465V and c.A1588C/p.(Asn530His)/N530H) were acquired from GenScript USA Inc. WT and mutant cDNA sequences were fully re-sequenced before being used in experiments to confirm the variant and exclude the presence of any additional sequence alterations. cRNA was prepared using the T7 mMessage kit from Ambion according to the manufacturer’s *instructions.*

### Electrophysiology

*Xenopus laevis* oocytes we received as a gift from the Department of Animal Physiology, Tübingen, Germany (1 mg/mL type CLS II collagenase, Biochrom, Berlin, Germany in OR-2 solution in mM: 82.5 NaCl, 2.5 KCl, 1 MgCl_2_ and 5 HEPES, pH7.5) or collagenase-treated *Xenopus laevis* oocytes were acquired from Ecocyte Bioscience, Dortmund, Germany, washed three times and stored at 16°C in Barth solution (in mM: 88 NaCl, 2.4 NaHCO_3_, 1 KCl, 0.33 Ca (NO_3_)_2_, 0.41 CaCl_2_, 0.82 MgSO_4_ and 5 Tris–HCl, pH7.4 with NaOH) supplemented with 50 μg/mL gentamycin (Biochrom). To compare current amplitudes of WT and mutant channels, 70 nL of cRNA encoding WT or mutant *KCNC2* cRNA (*c* = 1 μg/μl) were injected on the same day using the same batch of oocytes. For recordings of homozygous conditions of the WT or mutant K_V_3.2 channels 70 nL cRNA were injected. To be able to record the heterozygous condition, 35 nL of the WT and 35 nL of the related mutant *KCNC2* cRNA were injected. Injection was performed using the automated Roboinject system (Multi Channel Systems, Reutlingen, Germany). After 5 days of incubation at 17°C, potassium currents of injected oocytes were recorded at room temperature (20–22°C) using Roboocyte2 (Multi Channel Systems). For two-electrode voltage-clamp (TEVC) recordings, oocytes were impaled with two glass electrodes (resistance of 0.4–1MΩ) containing a solution of 1 M KCl and 1.5 M potassium acetate and clamped at a holding potential of −80 mV. Oocytes were perfused with ND96 bath solution containing (in mM): 93.5 NaCl, 2 KCl, 1.8 CaCl_2_, 2 MgCl_2_ and 5 HEPES (pH7.6). Currents were sampled at 2 kHz.

For the recordings with valproic acid (VPA) potassium currents in oocytes were initially perfused with ND96 Barth solution. After the first recording two short pulses (1 s) were used before the oocytes were perfused for 5 min with ND96 Barth solution containing 30 mM VPA. Subsequently, the potassium currents have been recorded again.

***Data and statistical analysis.*** Data analysis and graphical illustrations were achieved using Roboocyte2+ (Multichannel Systems, Germany), Excel (Microsoft, United States) and GraphPad Prism Software (GraphPad Software, United States). Normality was tested using the Shapiro–Wilk test and statistical evaluation for multiple comparisons was conducted using one-way ANOVA on ranks with Dunn’s *post hoc* test. Statistical testing was performed with SigmaPlot 12.0 (Systat Software, Inc.) and differences between groups were considered significant with *p < 0.05. Data are reported as mean ± SEM (standard error of the mean).

## Results

### Patients

All patients reported here have already been briefly presented by Schwarz and colleagues (9), but are described in more detail here. The patient (female, spontaneous birth) with the maternally inherited variant S333T presented with developmental and epileptic encephalopathy (DEE/Dravet-like). The mother suffers neither from epilepsy nor from febrile convulsions. No (even slight) cognitive deficits were found either. The patient has no other siblings. The age of the patient’s onset was 2 years and started with myoclonic seizures. At the age of 3 years they changed to generalized tonic–clonic seizures (GTCS). Fever was reported as a trigger for these seizures. EEG recordings showed generalized discharges at the age of 4 years. Brain MRI was unremarkable. This patient showed a mild intellectual disability, with delayed development. The ability to sit was acquired at the age of 6 years, to stand at the age of 7 and to walk at the age of 10. She was able to speak single words at the age of 7 years. The patient became seizure free using VPA. The patient is now 22 years old and receives 15 mg/kg VPA (plasma level 30 μg/L).

The patient (male, spontaneous birth) with the *de novo* variant F382C presented with myoclonic-atonic epilepsy (MAE). The seizures started at the age of 1 year with myoclonic, clonic and atonic seizures. Fever and fatigue were reported as a trigger for the seizures. Initial EEG (under fever) showed brief periods of theta-delta activity, repeatedly interrupted by paroxysms of high-amplitude delta waves and irregular spike–waves, two times pronounced myoclonias, later continuous irregular spike–wave pattern without recognizable seizures. MRI was normal except for a developmental right parietal venous anomaly. Intellectual disability (ID) or developmental delay could not be determined. Sulthiame (STM) had been administered in the past with no effect. To date, the patient is drug resistant and is no longer receiving antiseizure medication.

The patient (male) carrying the I465V variant presented with focal to bilateral tonic–clonic seizures (FBTCS). The mode of inheritance is unclear (father had febrile convulsions but was never genetically tested). Onset of seizures was at the age of 13 months. Initial EEG recordings revealed independent bilateral temporal lobe seizures. MRI was unremarkable, in particular no lesion could be detected in the temporal lobe. In the past, 15 different antiepileptic drugs were tried, but no seizure freedom could be achieved so far. Vagus nerve stimulation had no effect.

The variant N530H has been identified in a patient (female, spontaneous birth) with genetic generalized seizures (GGE), but the mode of inheritance is unclear (variant not present in mother, gDNA of unaffected father not available; father deceased). The age of onset was 8 years with generalized tonic–clonic seizures. At 25 years of age, two myoclonic seizures occurred. The patient has no intellectual disability or developmental delay but suffers from depression and anxiety. First EEG at the age of 24 years showed generalized 3 Hz spike-waves. No MRI has been performed. Valproic acid, Topiramate and Clobazam are currently administered with seizure reduction, but seizure freedom could not be achieved.

### Electrophysiological evaluation

We investigated four variants (see Table 1) according to the different phenotypes of the patients. These variants included one pathogenic variant (F382C) causing myoclonic-atonic epilepsy and three variants of uncertain significance (S333T, I465V and N530H - maternal inheritance or unknown mode of inheritance) causing different phenotypes such as focal epilepsy, genetic generalized epilepsy and developmental and epileptic encephalopathy.

**Table 1:** Data are presented as mean ± SEM, *k* slope factor.

	WT	S333T hom.	S333T het.	I465V hom.	I465V het.	N530H hom.	N530H het.	F382C hom.	F382C het.
Current density	1 ± 0.6	1.03 ± 0.19	1.06 ± 0.18	**0.07 ± 0.01**	0.54 ± 0.08	**1.33 ± 0.22**	0.86 ± 0.13	**0.14 ± 0.06**	**0.59 ± 0.1**
*n*	47	17	13	17	15	12	18	27	23
Activation									
V1/2	0.58 ± 0.56	**−1.61 ± 0.51**	0.33 ± 0.45	-	0.88 ± 1.89	0.91 ± 0.57	0.04 ± 0.46	-	**−4.98 ± 0.66**
*k*	−5.11 ± 0.11	−4.77 ± 0.08	**−4.64 ± 0.08**	-	**−5.91 ± 0.20**	−5.13 ± 0.15	−5.04 ± 0.17	-	**−5.84 ± 0.12**
*n*	47	17	13	-	15	12	18	-	23
Deactivation									
tau (0 mV)	54.56 ± 2.96	47.75 ± 2.51	62.70 ± 7.44	-	**15.36 ± 1.20**	52.16 ± 6.25	**83.83 ± 8.65**	-	**82.24 ± 15.32**
*n*	46	17	13	-	15	12	17	-	20

Significant differences are marked in bold.

**Figure 1:**
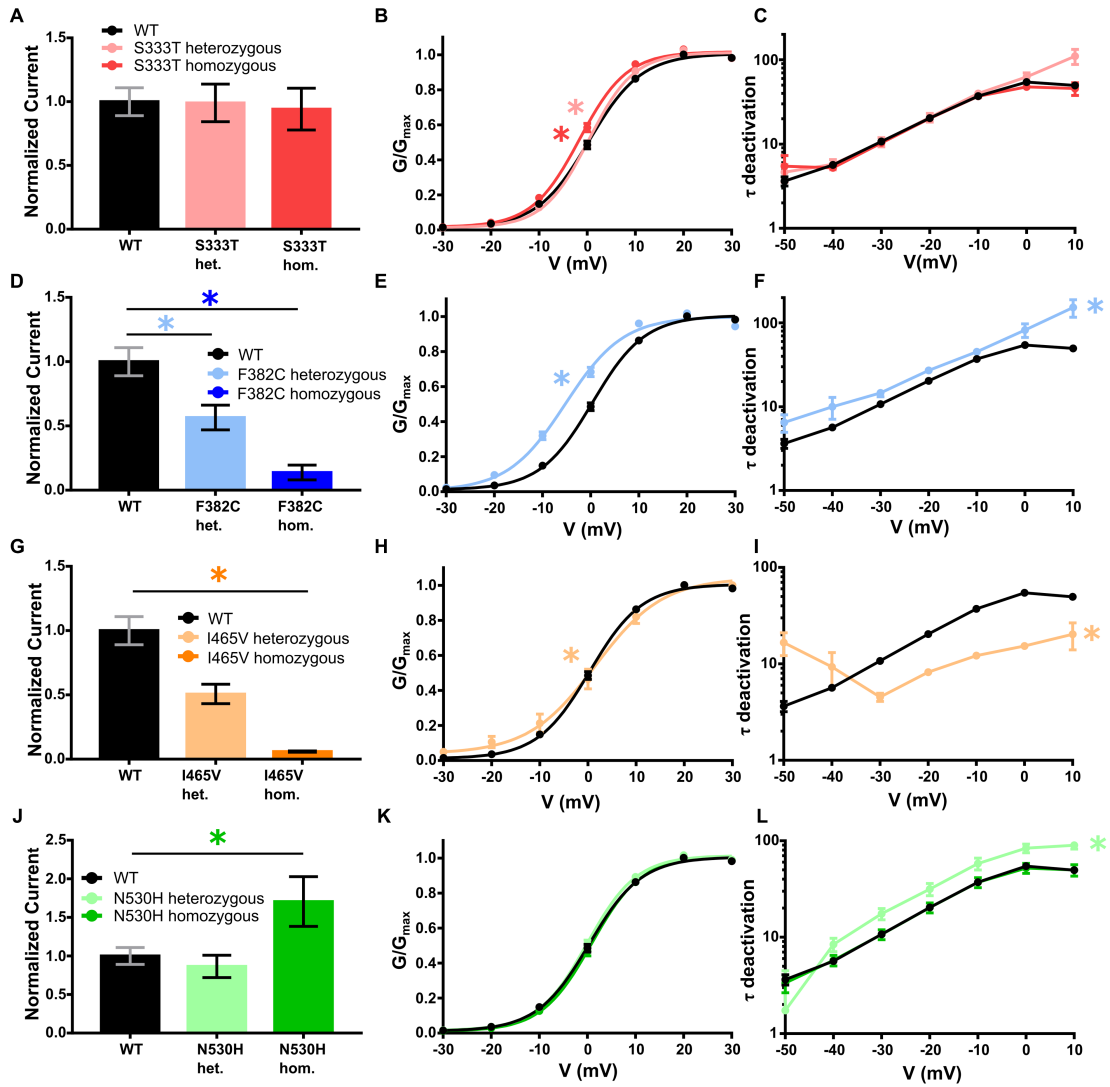
Electrophysiological analysis of the *KCNC2* variants F382C, I465V, N530H and S333T. **(A)** Mean current amplitudes of oocytes injected with WT (*n* = 47), S333T (*n* = 17) and equal amounts of WT + S333T (*n* = 13). **(B)** Mean voltage-dependent activation of K_V_3.2 channels for WT (*n* = 47), S333T (*n* = 17) and equal amounts of WT + S333T (*n* = 13). Lines illustrate Boltzmann Function fit to the data points. **(C)** Mean voltage-dependent deactivation time constant of K_V_3.2 channel WT (*n* = 46), S333T (*n* = 17) and WT + S333T (*n* = 13). **(D)** Mean current amplitudes of oocytes injected with WT (*n* = 47), F382C (*n* = 27) and equal amounts of WT + F382C (*n* = 23). **(E)** Mean voltage-dependent activation of K_V_3.2 channel for WT (*n* = 47) and equal amounts of WT + F382C (*n* = 23). Lines illustrate Boltzmann Function fit to the data points. **(F)** Mean voltage-dependent deactivation time constant of K_V_3.2 channel WT (*n* = 46) and WT + F382C (*n* = 20). **(G)** Mean current amplitudes of oocytes injected with WT (*n* = 47), I465V (*n* = 17) and equal amounts of WT + I465V (*n* = 15). **(H)** Mean voltage-dependent activation of K_V_3.2 channel for WT (*n* = 47) and equal amounts of WT + I465V (*n* = 15). Lines illustrate Boltzmann Function fit to the data points. **(I)** Mean voltage-dependent deactivation time constant of K_V_3.2 channel WT (*n* = 46) and WT + I465V (*n* = 15). **(J)** Mean current amplitudes of oocytes injected with WT (*n* = 47), N530H (*n* = 12) and equal amounts of WT + N530H (*n* = 18). **(K)** Mean voltage-dependent activation of K_V_3.2 channel for WT (*n* = 47), N530H (*n* = 12) and equal amounts of WT + N530H (*n* = 18). Lines illustrate Boltzmann Function fit to the data points. **(L)** Mean voltage-dependent deactivation time constant of K_V_3.2 channel WT (*n* = 46), N530H (*n* = 12) and WT + N530H (*n* = 17). All data are shown as means ± SEM. Statistically significant differences are indicated by an asterisk (*p < 0.05).

The S333T variant was maternally inherited, but without any maternal phenotype. The patient presented with DEE. This variant showed no change in the current density in either heterozygous or homozygous expression. The deactivation time constant was also unchanged. For the heterozygous expression the slope of the activation curve was significantly altered (−4.6) while the homozygous expression showed a shift of the half maximal voltage to a more hyperpolarized potential (−1.6 mV). Thus, this variant shows a gain-of-function (Table 1 and Figures 1A–C).

Heterozygous expression of the *de novo* variant F382C in *Xenopus laevis* oocytes resulted in a significant current reduction, without showing a dominant negative effect on the wildtype subunit. The activation kinetic of the heterozygously expressed channel showed a significant hyperpolarizing shift of 5 mV, the deactivation time-constant (τ) was significantly reduced at all recorded voltages (−50 to +10 mV). Homozygous expression of the F382C variant caused a dramatic reduction of the current amplitude, making it impossible to analyze the activation or the deactivation kinetics. Taken together, the variant F382C showed mixed changes in channel kinetics with gain-of-function changes in activation and deactivation and a clear loss-of-function in the current amplitude (Table 1 and Figures 1D–F).

The variant I465V had an unclear mode of inheritance. The expression of wildtype and mutant subunits showed a reduced current amplitude without any dominant negative effect. Analysis of the activation curve revealed a change in the slope factor (−5.9). Additionally, the deactivation time constant was significantly reduced. Homozygous expression of this variant showed a dramatic reduction of the current amplitude compared to WT. Thus, the activation and deactivation could not be analyzed (Table 1 and Figures 1G–I).

For the variant N530H the mode of inheritance was also unclear. The expression of the heterozygous state of this variant showed a similar current amplitude compared with the WT. In addition, the activation kinetic was not altered. However, the deactivation time constant was significantly increased for mutant channels compared with WT. In the homozygous state this variant showed an elevated current amplitude compared to WT, but neither the activation curve nor the deactivation time constant (τ) was altered (Table 1 and Figures 1J–L).

**Figure 2:**
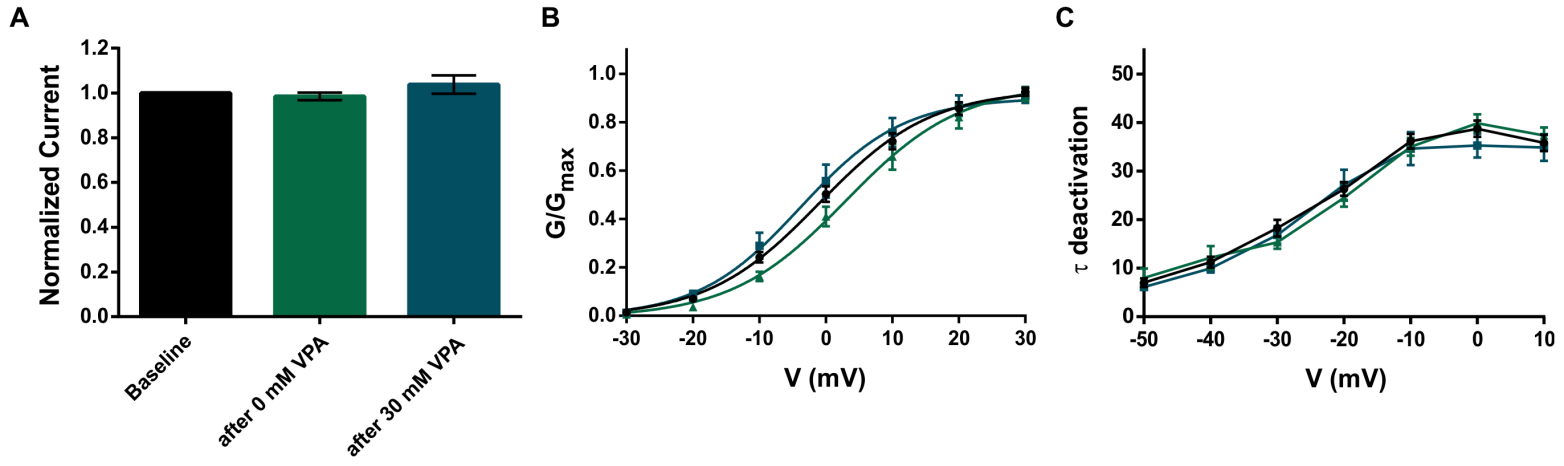
Application of 30 mM VPA does not affect K_V_3.2 WT channels. **(A)** Mean current amplitudes of oocytes injected with WT and recorded as baseline (*n* = 42), after perfusion with bath solution containing 0 mM VPA (*n* = 17) and after perfusion with bath solution containing 30 mM VPA (*n* = 12). **(B)** Mean voltage-dependent activation of K_V_3.2 channels for WT (*n* = 47), after 0 mM VPA (*n* = 17) and after 30 mM VPA (*n* = 12). Lines illustrate Boltzmann Function fit to the data points. **(C)** Mean voltage-dependent deactivation time constants of K_V_3.2 channels for WT (*n* = 47), after 0 mM VPA (*n* = 17) and after 30 mM VPA (*n* = 12). All data are shown as means ± SEM.

Recently, we reported that 8 out of 18 patients became seizure-free using VPA as monotherapy or in combination (9). Therefore, we wanted to investigate if and how valproic acid might affect the K_V_3.2 channel. We studied the effect of 30 mM valproic acid on the K_V_3.2 WT channel, but the recordings revealed neither an effect on the normalized current amplitudes nor on activation and deactivation (Figures 2A–C).

## Discussion

Here, we describe that also variants of uncertain significance in *KCNC2* may also be the underlying genetic etiology for different epilepsy syndromes. Previously, we described 18 patients harboring pathogenic variants or variants of uncertain significance in the *KCNC2* gene but the functional proof of the latter has not been performed yet (9). We have functionally investigated four more of these variants of unclear significance to show their effect in different phenotypes (MAE) and to analyze their potential pathogenic effect (DEE, GGE and FE). Therefore, we investigated one variant which was inherited from the healthy mother from a patient with DEE (S333T), one *de novo* variant from a patient with MAE (F382C) and two variants with unknown mode of inheritance from patients with focal epilepsy (I465V) and GGE (N530H). S333T showed gain-of-function effects in the activation kinetics in both the heterozygous and homozygous state. However, due to the unaffected mother, we classify this variant as a modifying variant with incomplete penetrance. The challenge in modifying variants with incomplete penetrance is to understand the complex interplay of genetic and environmental factors that determine an individual’s phenotype. Therefore, an analysis on a model system with a respective genetic background (e.g., iPSC-derived neurons) from both the healthy mother and the affected patient would be beneficial to possibly better explain the phenotype.

Electrophysiological analysis showed a mixed effect (gain-of-function changes in activation and deactivation and a clear loss-of-function in the current amplitude) but overall, it is more likely to be loss-of-function for the *de novo* variant F382C which was found in a patient with MAE. This is a similar finding as previously described for the variant T437A which was found in a patient with early-onset absence epilepsy (EOAE) (9). Both patients are drug resistant and show a generalization in the EEG, but there are also some differences in the phenotypes. Seizure types and the development differed between the patients, with absence and tonic seizures and a severe developmental delay in the EOAE patient carrying the T437A variant and myoclonic, atonic and clonic seizures without developmental delay in the patients with the F382C variant. The similar, but still not exactly the identical, pathophysiological effects could be due to adjacent spatial proximity of the two variants, as predicted by Alphafold (12, 13), and therefore lead to similar functional changes in the channel upon replacement of the corresponding amino acid.

The other two variants tested here were declared as likely pathogenic because the family history of the patient with the I465V variant showed febrile seizures in the father and in the first grade cousin, which might suggest that the variant is inherited, but we could not prove this further due to the unavailability of samples. This variant showed a significant reduction in the current amplitude, a change in the slope factor of the activation of the channel and an accelerated deactivation constant thus causing a clear loss-of-function.

The family history of the other variant with unclear mode of inheritance, N530H, was negative for the mother (gDNA of unaffected father not available; father deceased). This variant showed an increased current amplitude in the homozygous state, while there was no change in current amplitude in the heterozygous state. This could mean that the variant affects the transport of the channel to the membrane, since the C-terminus is an important interaction site for Ankyrin G, which helps to guide the channel to the membrane (14). Surprisingly, this effect seems to be reversed in the heteromeric state. Further studies are needed to clarify the potential effect of this variant on channel localization and the interaction with Ankyrin G as well as the T1 domain which is present in all K_V_2, K_V_ 3, and K_V_ 4 channels and is required for tetramerization of K_V_ subunits within the same subfamily (15). In addition, the T1 domains also play an important role in the interaction of the channel with cytoplasmic regulatory β-subunits (K_V_β) (16).

The previously described patient carrying the S333T variant, as well as other cases presented in Schwarz and colleagues (9), responded well to VPA. Therefore, we wanted to further investigate possible effects of VPA on the K_V_3.2 channel. Although the mechanism of action remains poorly understood, VPA has been used for decades in the treatment of epilepsy (17). The anticonvulsant effect is attributed in part to the blockade of voltage-gated sodium channels and the increase in gamma-aminobutyric acid (GABA) levels in the brain (18, 19). But VPA also has various effects on potassium channels (20, 21), such as maintaining the M-current, a low-threshold non-inactivating potassium current, during seizures (22). However, in our recordings, we could not observe any effect of VPA on the potassium channel K_V_3.2. Therefore, effects on other proteins are likely to play a more important role in achieving seizure freedom in patients.

In this work, we have reported the functional consequences of four variants in the *KCNC2* gene, which has recently been reported as a novel epilepsy gene (9). We could demonstrate that also variants of uncertain significance in *KCNC2* classified by the ACMG criteria can have a significant effect on the channel function and therefore be associated to different epilepsy phenotypes. Unfortunately, we could not explain the beneficial effect of VPA in several described patients by an effect of the drug on the K_V_3.2 channel. Again, more complex model systems such as iPSC-derived neurons from affected patients with appropriate expression of different ion channels may be needed to elucidate the underlying mechanism.

## Data availability statement

The raw data supporting the conclusions of this article will be made available by the authors, without undue reservation.

## Author contributions

SS and MP: drafting and revision of the manuscript for content, including medical writing for content, major role in the acquisition of data, and analysis or interpretation of data. IH, YW, and NS: drafting and revision of the manuscript for content, including medical writing for content, study concept or design, and analysis or interpretation of data. All authors contributed to the article and approved the submitted version.

## Funding

We acknowledge support by the Open Access Publishing Fund of University of Tübingen. The study also received support through the DFG/FNR INTER Research Unit FOR2715 (WE 4896/4-2 to YW). NS and YW were supported by the BMBF Treat-ION grant (01GM2210B).

## Conflict of interest

The authors declare that the research was conducted in the absence of any commercial or financial relationships that could be construed as a potential conflict of interest.

## Publisher’s note

All claims expressed in this article are solely those of the authors and do not necessarily represent those of their affiliated organizations, or those of the publisher, the editors and the reviewers. Any product that may be evaluated in this article, or claim that may be made by its manufacturer, is not guaranteed or endorsed by the publisher.
